# Health and socioeconomic well-being of women with endometriosis and provoked vestibulodynia: Longitudinal insights from Swedish registry data

**DOI:** 10.1371/journal.pone.0307412

**Published:** 2024-09-03

**Authors:** Hanna Mühlrad, Matts Olovsson, Evelina Linnros, Philip Haraldson, Nina Bohm-Starke

**Affiliations:** 1 Department of Clinical Sciences, Danderyd Hospital, Karolinska Institutet, Stockholm, Sweden; 2 Department of Women’s and Children’s Health, Uppsala University, Uppsala, Sweden; 3 Institute for International Economic Studies, Stockholm University, Stockholm, Sweden; Iranian Institute for Health Sciences Research, ISLAMIC REPUBLIC OF IRAN

## Abstract

Endometriosis and provoked vestibulodynia (PVD) are prevalent pain conditions among women of reproductive age, significantly impacting their quality of life and psychological well-being. However, comprehensive evidence regarding the lifelong health and socioeconomic outcomes for these individuals remains scarce. Additionally, many prior studies rely on limited and sometimes unrepresentative samples. This study aims to inform on the long-term consequences of these disorders by examining health, fertility, and employment outcomes in a cohort of women diagnosed with endometriosis and/or PVD, tracing their experiences from childhood to their 40s. Leveraging nationwide administrative data from Sweden and employing a matched case-control design, we investigate both similarities and differences between women with these diagnoses and those without. Our findings indicate that women diagnosed with endometriosis and/or PVD demonstrate elevated healthcare utilization patterns, commencing in their early teenage years and progressively increasing over time. Notably, disparities in labor market outcomes emerge in their 20s, showcasing lower labor earnings and a rise in sickness benefit receipt. Moreover, our results show a higher likelihood among these women to experience mental health disorders and concurrent chronic pain diseases, as well as infertility. While the association between endometriosis and infertility is well-documented, this study offers novel insights into a potential similar link between PVD and infertility. Our study informs healthcare professionals and policymakers about the considerable burden of compromised health, adverse psychosocial well-being, and reduced productivity in the labor market faced by young women with these common pain conditions. These findings underscore the urgency of addressing the multifaceted challenges encountered by individuals diagnosed with endometriosis and PVD across their lifespan.

## Introduction

Health is essential to an individual’s well-being and their human capital. Chronic pain disorders are prevalent and present significant challenges for individuals and societies in terms of high healthcare costs, reduced labor market productivity, and long-term disability [1–3]. In the United States alone, costs associated with chronic pain are estimated to range between 560–635 billion USD per year [4, 5]. In Europe, chronic pain conditions are estimated to account for as much as 3–10% of GDP [6].

Research indicates that chronic pain is more prevalent in women than in men, with conditions such as migraines, irritable bowel syndrome, and fibromyalgia being more common in women [7–9]. In addition, women are also more likely to experience chronic pelvic pain conditions. Two common pain conditions in women are endometriosis and provoked vestibulodynia (PVD). These diagnoses affect an estimated 10% and 8% of all women of fertile age, respectively [4, 10].

Although endometriosis and PVD have different pain characteristics and pathophysiology, they share several features that have a significant impact on quality of life and psychosocial health [11, 12]. Endometriosis is characterized by severe dysmenorrhea and dyspareunia, but non-menstrual pelvic pain/cramping and other debilitating symptoms may also be present [13]. Women with PVD on the other hand suffers from an intermittent pain, mainly provoked by vaginal intercourse or tampon insertion [14]. Both conditions are also associated with delay in diagnosis, a high prevalence of mental health issues and concurrent pain conditions [15–20]. Moreover, endometriosis is associated with higher risk of opioid dependence/abuse [21].

Chronic pain disorders generate high utilization of medical care and result in a substantial economic burden for both individuals and society. Estimates of the direct costs of endometriosis range from approximately 1100 USD per patient per year in Canada to 12 000 USD in the US [22]. Corresponding annual costs for endometriosis reported in a Swedish survey are estimated to 4,282 EUR for direct and 4,486 EUR for indirect costs [23]. Previous research on direct costs for in- and outpatient care for PVD is limited, but a survey in the US estimated the total cost during six months to be 8862 USD per woman, of which 68% was direct healthcare costs and 26% indirect costs [24].

Moreover, endometriosis is associated with a higher risk of infertility [25–27]. Research has shown that infertility in women with endometriosis is linked to anatomical distortions from adhesions and fibrosis, disturbances in both the ovarian- and endometrial function, and endocrine- and immunological systems [28–30]. Less is known about fertility outcomes in women with PVD. However, a previous study has documented that women diagnosed with PVD tend to exhibit lower fertility [31], and a report from the Swedish National Board of Health and Welfare has shown a higher reliance on assisted reproductive treatments, such as in-vitro fertilization (IVF), compared to mothers without PVD. Although the mechanisms behind this association remain unknown, it is plausible that factors such as dyspareunia and underlying health conditions—more prevalent in women with PVD, including immune dysfunction disorders—might play a role [32].

Other life aspects affected by chronic pain conditions are reduced labor market productivity from suboptimal human capital investments, sickness absence and higher social insurance uptake. Previous studies, based mainly on survey data, suggest that women suffering from endometriosis have impaired educational attainment and poorer labor market productivity with indirect costs from lost work productivity ranging from 3 300 USD per patient/year in Austria to 15 700 USD in the US [22].

Despite the high prevalence of endometriosis and PVD, there is a lack of evidence on the long-term implications of these diagnoses for health and socioeconomic well-being. Previous research has investigated concurrent health issues, potential consequences, high healthcare utilization, and diminished quality of life associated with these diagnoses. However, there remains a significant gap in the literature regarding comprehensive analyses spanning the entire reproductive period.

To the best of our knowledge, systematic evidence on the direct and indirect costs associated with endometriosis and PVD derived from administrative data is limited. Furthermore, there is a paucity of research specifically addressing the longitudinal aspects of healthcare utilization and labor market outcomes over time in relation to these conditions.

To address this knowledge gap, we utilize nationwide longitudinal registry data from Sweden and employ a case-control study design to examine:

1. The prevalence of comorbidities, including mental health disorders, concurrent pain disorders, and infertility.
2. The evolution of health utilization and labor market productivity over time.

Using the rich Swedish administrative data, we link longitudinal information about health and labor market outcomes, following individuals from childhood to adulthood. Thus, our study aims to provide valuable insights into the development of health and socioeconomic well-being among women diagnosed with endometriosis and/or PVD.

## Materials and methods

### Data sources and study design

In Sweden, residents are assigned a unique personal identification number (at birth or upon moving to Sweden), which allows for the linkage of data from multiple Swedish registries, including those maintained by the Swedish National Board of Health and Welfare and Statistics Sweden. Our study received approval from the Swedish Ethical Review Authority (reference number: 2017/299-31).

Medical information on dysmenorrhea, vulvar and pelvic pain, comorbidity, and infertility was obtained from the Swedish National Patient Registry (NPR), which contains data on diagnosis, admission and discharge dates, procedures, and treatments. Obstetric data was obtained from the Medical Birth Registry (MBR), while labor market information ‐ including income from gainful employment, sickness and unemployment benefits, and highest educational attainment ‐ was sourced from the Longitudinal Integration Database for Health Insurance and Labor Market Studies (LISA, Statistics Sweden). LISA contains annual information on labor market outcomes for individuals aged 16 years and older, starting from 1990. Data from NPR and MBR was collected until 2018, while information from LISA was collected until 2016.

### Study population

Our sample of cases and controls consisted of women born in Sweden between 1973–1996, registered in the MBR, and were identified using the NPR for the years 1997–2018. We used a matched case-control design, with two controls for each woman born in Sweden 1973–1996 with diagnosed endometriosis and/or PVD during 1997–2018. The matching criterion was based on age, being born in Sweden, being alive and living in Sweden until 2018 such that women emigrating from Sweden or dying before 2018, were excluded from this analysis. Our cases of endometriosis and PVD were diagnosed according to the ICD-10 system. The diagnosis of endometriosis is based on physical examination, imaging technologies such as ultrasound and magnetic resonance imaging (MRI) and laparoscopy [33]. In this study, the codes N80.1-N80.9 and N97.8D were used for endometriosis. No physiological markers exist for diagnosing vulvodynia. The diagnosis is made by the patient’s history of pain at contact of the vestibular mucosa and physical examination excluding other causes of vulvar pain [34]. In the registers, the code N76.3 was used for provoked vestibulodynia and additionally as N94.2 and F52.5 for vaginismus (as this condition is oftentimes accompanying provoked vestibulodynia to some degree) [31].

Our cases consist of three groups. The first group consists of women with diagnosed endometriosis (n = 18414) and the second group of women with diagnosed vulvodynia (n = 9752). The third group consists of women diagnosed with both endometriosis and PVD (n = 565). The control group consists of two randomly selected women per case, matched by birth year, who were not diagnosed with endometriosis or vulvodynia (n = 57462) during the period 1997–2018.

### Outcomes of interest

Demographic information on birth year, marital status (legally married during at least one year) and highest educational attainment was collected from LISA registry for the period 1990–2016. We consider diagnosed infertility defined by ICD-10 code N97. Using the MBR, for a subset of women having at least one pregnancy carried beyond 22 weeks of gestation (both alive and stillbirths), we construct a binary variable for the use of in-vitro fertilization (both standard IVF and intracytoplasmic sperm injection (ICSI)). The information on the number of births comes from the MBR. We examine the occurrence of comorbidities, identified via the NPR for the period 1997–2018 with diagnose codes set according to the ICD-10. The comorbidities are analyzed in subcategories; a composite index of pain disorders (chronic headaches and migraine, gastrointestinal, muscles and joints, bladder), a composite index of psychiatric disorders (depression, recurrent depression, anxiety, obsessive and compulsive, stress reaction, eating and sleeping disorders), substance abuse and neuropsychiatric disorders. A detailed description of included ICD-codes is provided in S1 Fille (supplementary information). Health care utilization is measured by the numbers of in-patient and out-patient visits (which is based on the NPR for the period 1987–2018 for in-patient visits and 2001–2018 for out-patient visits). Finally, we examine labor market outcomes across cases and controls, using information on annual income from wage earnings, sickness benefits, and unemployment benefits obtained from LISA for the period 1990–2016. We report income at each age as well as the average income.

### Statistical analysis

Differences in comorbidity, health care utilization, fertility outcomes, and labor market productivity are analyzed using logistic regression for binary outcomes and linear regression using the ordinary least squares method for continuous outcomes. Because age is likely a strong confounder for our outcomes, we adjust for age fixed effects in all regressions, as proposed by Pearce [35]. The idiosyncratic standard errors are clustered on the level of matching ID-number. We present adjusted odds ratios (aOR) and 95% confidence intervals (CI) along with p-values. Because we consider multiple outcomes, the analysis is susceptible to Type-I errors. To account for this potential issue, we conduct a multiple-comparison correction according to Bonferroni and present an indicator for insignificant findings (P-value >0.05). As educational attainment is likely an outcome variable that could be affected by endometriosis and PVD, we do not control for this factor as it may introduce bias [36].

To examine labor market trajectories and healthcare visits over time, we use a linear regression with individual fixed effects to estimate the differences in outcomes between our groups (endometriosis and PVD) and the control population, shown in coefficient plots. The data analysis was conducted using STATA 14.0 (StataCorp, College Station, Texas, USA).

## Results

### Main results

**Fig 1:**
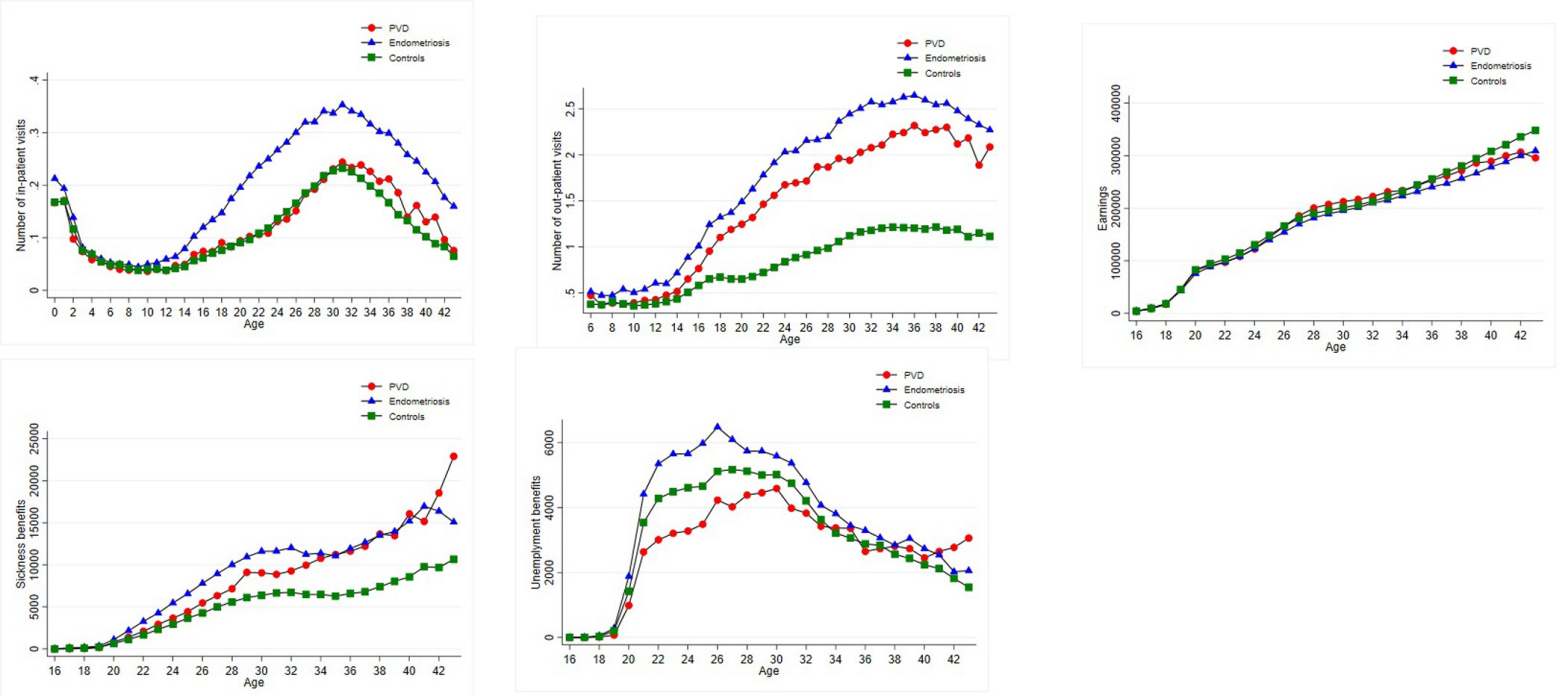
Results from descriptive raw data. (a) In-patient visits (b) Out-patient visits (c) Wage earnings (SEK) (d) Income from sickness benefits (SEK) (e) Income from unemployment benefits (SEK). Number of inpatients visits, ages 0–43. Number of out-patient visits, ages 6–43. Labor market outcomes, ages 16–43. More detailed information on outcome trajectories for all groups including endometriosis and/or PVD can be found in S1A–S1E Fig in the Supplementary information.

Summary statistics are presented in Table 1 and Fig 1, S1 Fig for women with endometriosis, PVD, both endometriosis and PVD, and the control group. Means and proportions were used to illustrate the characteristics and outcomes in women diagnosed with endometriosis and/or PVD, as well as in women without these conditions. Additionally, results from linear and logistic regressions, examining differences in outcomes across women with and without diagnosed PVD and/or endometriosis, are presented in Table 2.

**Table 1:** Patient characteristics and outcome.

	Endometriosis	PVD	PVD and endometriosis	Controls
	n = 18414	n = 9752	n = 565	n = 57462
**Birth year** (mean)	1982	1985	1984	1983
**Married** (%)	28%	46%	40%	35%
**Fertility**				
Number of children (mean)	1.12	0.86	0.88	1.23
Involuntary infertility	29%	9%	27%	7%
IVF (of women with pregnancy beyond 22 weeks)	21%	5%	18%	4%
**Comorbidity (%)**				
***Pain disorders (composite index)***	44%	37%	60%	24%
Migraine	22%	17%	27%	12%
IBS	8%	6%	13%	2%
Back pain	23%	16%	30%	11%
Urethritis	2%	4%	9%	1%
Unspecified pain	14%	8%	22%	4%
***Psychiatric conditions (composite index)***	32%	31%	43%	19%
Depression	19%	18%	25%	10%
Anxiety	19%	19%	29%	10%
OCD	2%	3%	3%	1%
Stress reactions	14%	11%	19%	7%
Eating disorder	4%	4%	5%	3%
Sleeping disorder	3%	3%	4%	1%
***Substance abuse***	2%	1%	1%	1%
***Neuropsychiatric disorders***	6%	6%	6%	4%
**Mean number of annual in-and outpatient visits**				
Inpatient visits (hospital nights)	0.19	0.11	0.20	0.11
Outpatient visits	2.03	1.50	2.70	0.90
**Educational attainment (%)**				
Elementary	7%	3%	5%	6%
High school	42%	32%	33%	40%
University	51%	65%	63%	54%
Missing information	35	13	-	367
**Annual income at age 35 (SEK)**				
Wage earnings	231 689	242 895	228 247	243 978
Sickness benefits	11 054	11 225	16 129	6 284
Unemployment benefits	3 231	3 178	2 080	2 870

PVD, provoked vestibulodynia; IVF, in vitro fertilization; IBS, irritable bowel syndrome; OCD, obsessive compulsive disorder; SEK, Swedish crown (Swedish currency, 1 SEK = 0.10 USD). For detailed information on ICD codes, see S1 File (Suppl. Information).

**Table 2:** Results from regression analyses.

	Endometriosis		PVD		PVD/Endometriosis		
**Fertility**	*aOR*	*95%CI*	*aOR*	*95% CI*	*aOR*	*95% CI*	*n*
Infertility	5.44***	[5.20,5.70]	1.49***	[1.38,1.61]	5.30***	[4.37,6.43]	86193
IVF birth	6.25***	[5.82,6.71]	1.37***	[1.19,1.58]	5.73***	[4.24,7.73]	50850
	*B*	*95%CI*	*B*	*95%CI*	*B*	*95%CI*	*n*
Number of children	-0.21***	[-0.23, -0.19]	-0.18***	[-0.20, -0.17]	-0.30***	[-0.37, -0.23]	86193
**Comorbidity**	*aOR*	*95%CI*	*aOR*	*95% CI*	*aOR*	*95% CI*	*n*
Pain disorders	2.56***	[2.47,2.65]	1.89***	[1.81,1.98]	4.78***	[4.03,5.66]	86193
Psychiatric disorders	2.05***	[1.97,2.12]	1.90***	[1.81,1.99]	3.18***	[2.68,3.76]	86193
Substance abuse	1.63***	[1.44,1.85]	0.75**⸸	[0.61,0.93]	0.95	[0.45,2.00]	86193
Neuro-psychiatric disorders	1.88***	[1.75,2.03]	1.47***	[1.33,1.62]	1.62**⸸	[1.13,2.31]	86193
**Annual in-and outpatient visits**	*B*	*95%CI*	*B*	*95%CI*	*B*	*95%CI*	*n*
Average in-patient visits	0.22***	[0.21,0.23]	0.01	[-0.01,0.02]	0.22***	[0.16,0.28]	86193
Average out-patient visits	1.24***	[1.21,1.27]	0.62***	[0.59,0.66]	1.84***	[1.64,2.04]	86193
	*aOR*	*95%CI*	*aOR*	*95% CI*	*aOR*	*95% CI*	*n*
**University education**	0.85***	[0.82,0.88]	1.73***	[1.65,1.81]	1.41***	[1.18,1.68]	85778
**Mean annual Income (SEK)**	*B*	*95%CI*	*B*	*95%CI*	*B*	*95%CI*	*n*
Wage earnings	-5785***	[–6868, –4702]	-3520***	[–4770, –2270]	-12641***	[–17482, –7800]	86188
Sickness benefits	2645***	[2486,2803]	1114***	[948,1280]	4233***	[3281,5184]	86188
Unemployment benefits	279***	[199,359]	7	[–77,90]	78	[–270,425]	86188

PVD, provoked vestibulodynia. IVF, in vitro fertilization. aOR, adjusted odds ratio. *b*, beta coefficient from ordinary least squares regression. 95% CI, confidence interval. n, number of observations.

* P <0.05

** P <0.01

*** P<0.001.

⸸ insignificant (P>0.05) when adjusting p-value for multiple hypothesis testing according to Bonferroni method. SEK, Swedish crown (Swedish currency, 1 SEK = 0.10 USD).

Our sample consisted of women born between 1973–1996, with the mean birth year ranging from 1982 to 1985 (Table 1). The proportion of women legally married for at least one year between 1990 and 2016 was 28% in women with endometriosis, 46% in women with PVD, 40% in women with both endometriosis and PVD, and 35% in the control group (Table 1).

Adverse health outcomes, including infertility, concomitant pain disorders, and higher rates of psychiatric and neuropsychiatric disorders, were more prevalent in women with endometriosis, PVD, or both, than in the control group (Table 1 and Fig 1A–1E). More specifically, we documented an elevated risk of infertility in women with endometriosis, PVD, or both (Table 2), with higher odds of being diagnosed with infertility compared to controls: aOR 5.4 (95% CI: 5.2–5.7) for endometriosis, aOR 1.5 (95% CI: 1.4–1.6) for PVD, and aOR 5.3 (95% CI: 4.4–6.4) for women with both endometriosis and PVD. For those with a pregnancy carried out beyond 22 weeks of gestation, women with endometriosis, PVD, or both had a higher aOR of IVF conception, 6.3 (95% CI: 5.8–6.7), 1.4 (95% CI: 1.2–1.6), and 5.7 (95% CI: 4.2–7.7), respectively. Moreover, the OLS estimates showed that women with endometriosis, PVD, or both gave birth to 0.2–0.3 fewer children compared to controls (Table 2). This corresponded to a negative impact on fertility by 15–24% when compared to the mean number of children in the control group.

Results from the logistic regressions showed higher aORs of concomitant pain disorders of magnitude, 2.6 (95% CI: 2.5–2.7) for women with endometriosis, 1.9 (95% CI: 1.8–2.0) for women with PVD, and 4.8 (95% CI: 4.0–5.7) for women with both endometriosis and PVD (Table 2). The aORs for psychiatric disorders were almost identical for women with endometriosis and PVD, with approximately a twofold higher odds ratio compared to controls, with an aOR of 2.0 (95% CI: 2.0–2.1) for endometriosis, 1.9 (95% CI: 1.8–2.0) for PVD, and 3.2 (95% CI: 2.7–3.8) for both. Compared to the control group, the odds for neuropsychiatric diagnoses were higher for women with endometriosis (aOR 1.9, 95% CI:1.7–2.0), PVD (aOR 1.5, 95% CI:1.3–1.6), or both (aOR 1.6, 95% CI: 1.1–2.3). An increased aOR of substance abuse was only found for women with endometriosis (aOR 1.6, 95% CI: 1.4–1.9).

In terms of healthcare utilization, women with endometriosis, PVD, or both had more healthcare visits compared to controls starting from around the age of menarche (Table 1 and Fig 1A). Compared to controls, women with endometriosis had 0.2 (95% CI: 0.2–0.2) more in-patient visits each year and 1.2 (95% CI: 1.2–1.3) more out-patient visits each year (Table 2). There was no difference in the number of annual in-patient visits between women with PVD and controls; however, women with PVD had 0.6 (95% CI: 0.6–0.7) more out-patient visits annually compared to controls. Similarly, women with both PVD and endometriosis had 0.2 more in-patient visits and 1.8 more out-patient visits each year.

The highest educational attainment was observed in the PVD group, with 65% having a university education, followed by women with both PVD and endometriosis at 63%, the control group at 54%, and the endometriosis group at 51% (Table 1). Compared to controls, women with PVD had a higher aOR of 1.7 (95% CI: 1.7–1.8) for having a university education, while women with endometriosis had a lower aOR of 0.85 (95% CI: 0.8–0.9), as reported in Table 2. Women with both endometriosis and PVD had a higher aOR for university education of 1.4 (95% CI: 1.2–1.7) compared to controls.

Women with endometriosis, PVD, or both had on average lower labor market productivity at age 35 (Table 1), with increasingly lower annual wage earnings and higher income from sickness benefits over time (Fig 1C–1E). Moreover, women with endometriosis also received more income from unemployment benefits compared to controls. The opposite pattern was observed for women with diagnosed PVD. More specifically, results from linear regressions (Table 2) suggest that annual average wage earnings were lower for women with endometriosis, PVD, or both, compared to controls by 5785, 3520, and 12641 SEK, respectively. The average annual income from sickness benefits was significantly higher for women with endometriosis, PVD, or both compared to controls by 2645, 1114, and 4233 SEK, respectively.

### Health and labor market trajectories

**Fig 2:**
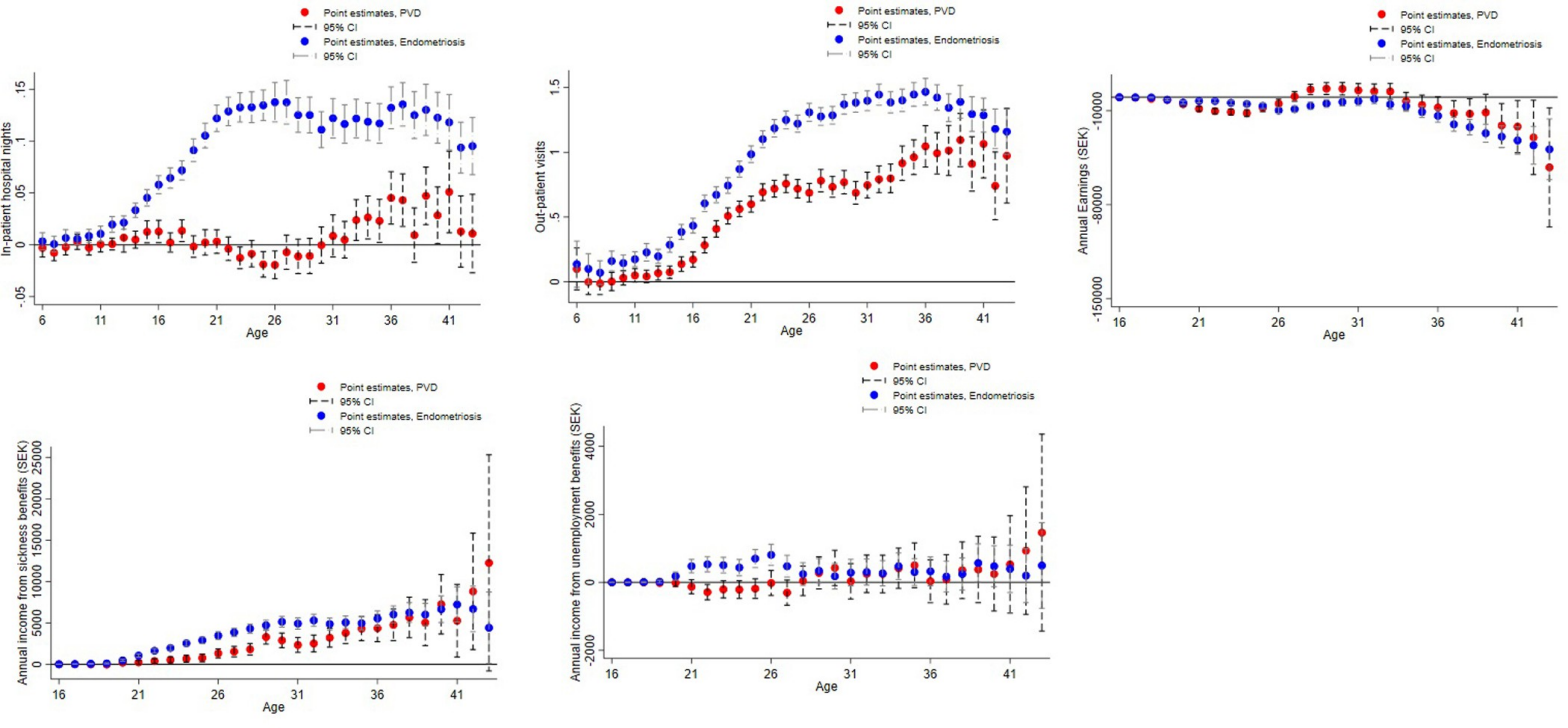
Results from regression analyses of in- and outpatient visits and labor market outcomes. (a) In-patient visits (b) Out-patient visits (c) Earnings (SEK) (d) Income from sickness benefits (SEK) (e) Income from unemployment benefits (SEK). Coefficient plots with point estimates and 95% confidence interval. Black line represents control women.

Fig 2A–2E display linear regression coefficients and 95% CI of how in-and out-patient visits and labor market outcomes evolve over women’s life course compared to controls from ages 0–43 for in-patient visits, ages 6–43 for out-patient visits, and 16–43 for labor market outcomes. These coefficient plots depict the difference between control women to women with endometriosis or PVD, for each age, separately. Coefficient plots estimating effects for women with endometriosis and PVD can be found in the appendix (S2A–S2E Fig).

Women with endometriosis had up to 0.12 more in-patient visits per year and approximately 1.4 more out-patient visits per year compared to controls (Fig 2A and 2B). The increase of in- and out-patient visits starts in early teens, which is the age around menarche. In-patient visits appear similar between women with PVD and controls until age 30, when a small increase is seen for women with PVD, Fig 2A. Around the age of menarche, women with PVD had up to one additional out-patient visit per year, Fig 2B. Women with both endometriosis and PVD had the highest number of both in- and out-patient visits (S1 Fig, Supplementary information).

Fig 2C–2E present labor market trajectories. Women with endometriosis and PVD exhibited consistently lower annual wage earnings and a slower growth rate in earnings compared to the controls. Women with endometriosis had persistently lower wage earnings by up-to 40 000 SEK per year (Fig 2C) and higher income from both sickness (by up to 10 000 SEK per year) and unemployment benefits (Fig 2D and 2E). Compared to controls, women with PVD earn up to 10 000 SEK less per year during ages 36–43 (Fig 2C) and had higher annual income from sickness benefits during their mid-twenties to mid-forties (Fig 2D). Labor market trajectories for women with both endometriosis and PVD are shown in S2C–S2E Fig, supplementary information.

## Discussion

### Principal findings

Using large nationwide medical records from Sweden, we assess the association between common pain conditions in women, endometriosis and PVD, and health and economic outcomes up to the age of 43. In this longitudinal study, we analyze registry data from nearly 19,000 women with endometriosis and over 10,000 women with PVD to investigate the association between these conditions and health and economic outcomes.

This study highlights the severe impact of endometriosis and PVD on women’s wellbeing by examining health and economic outcomes over time. Our study illustrates the significant burden that endometriosis and PVD place on the healthcare sector and labor market productivity, impacting both individuals and society at large. Our results further stress the need for developing and implementing targeted programs and efforts to improve access to treatment and support for women with endometriosis and PVD.

Consistent with previous studies [11, 19, 23] we found that women with endometriosis and/or PVD experience worse health outcomes, including a higher likelihood of infertility, concurrent pain, mental health, and neuro-psychiatric disorders, as well as a greater number of healthcare visits. Our study is the first to establish higher odds of diagnosed infertility in women with PVD, along with higher IVF usage and lower fertility. Moreover, women diagnosed with endometriosis and/or PVD demonstrate higher healthcare utilization, beginning as early as their teenage years, compared to controls. Our study represents the first investigation into the longitudinal patterns of healthcare utilization among women diagnosed with endometriosis and PVD.

In line with previous studies suggesting productivity loss in populations with chronic pain disorders, our results suggest that women with endometriosis and/or PVD had consistently higher income from sickness insurance and persistently lower wage income over time. To the best or our knowledge, this is the first study to present health and labor market trajectories over the life course up to mid-forties for women with endometriosis and PVD.

### Policy and clinical implications

Our findings underline several significant policy implications concerning the adverse effects of chronic pain on women’s health and socio-economic well-being. Firstly, our results indicate nearly identical associations between pelvic/vulvar pain and the risk of depression and anxiety, regardless of the specific pain condition. This prompts inquiry into whether most chronic pain conditions in women exert similar impacts on mental health and labor market outcomes, irrespective of their root cause. Further research is essential to investigate the efficacy of healthcare programs and treatments for addressing mental health issues in improving overall well-being and labor market outcomes among women diagnosed with endometriosis and/or PVD.

Despite the increased attention given to endometriosis and PVD in recent years, there remains a considerable number of unrecognized cases [37]. Thus, early recognition of chronic pain conditions such as endometriosis and PVD are crucial for affected women to receive appropriate support and evidence-based treatments.

Our findings reveal a negative association between PVD and infertility, thus suggesting a potential need for enhanced family planning counseling for this patient group. For women coping with PVD, anecdotal evidence from clinicians indicates concerns about family formation due to difficulties with vaginal intercourse [38]. However, self-insemination at home, might offer an option for women with regular ovulations. Clinicians attending to these patients should provide comprehensive information and support. Additionally, IVF treatments in women with both endometriosis and PVD may pose challenges, as procedures like ultrasound monitoring, egg retrieval, and embryo transfer could induce severe vaginal and pelvic pain. Moreover, the limited availability of adequate anesthetics in many clinics could exacerbate this issue.

Finally, it is noteworthy that women diagnosed with endometriosis and/or PVD demonstrate a higher prevalence of neuro-psychiatric disorders such as autistic spectrum disorder (ASD) and attention-deficit/hyperactive disorder (ADHD). ASD has been linked to altered pain perception [39], potentially influencing treatment outcomes. Therefore, routine screening for these comorbidities is crucial within clinical settings. For instance, a longitudinal study revealed that women diagnosed with ASD and/or ADHD who received stimulant medications for their conditions showed a reduced incidence of chronic widespread pain compared to those not on medication [40]. Beyond potentially limited effectiveness in pain management, women with ASD/ADHD comorbidity might also exhibit a diminished response to psychological interventions commonly employed as part of the multi-disciplinary treatment approach for endometriosis and PVD [41, 42].

### Strengths and limitations

In this study, we utilize high-quality longitudinal registry data to investigate the association of endometriosis and PVD on health outcomes and labor market outcomes among a nationwide sample of over 28,700 women. Our use of longitudinal registry data provides a larger and more representative sample than previous studies, which oftentimes rely on surveys and may have suffered from recall bias. Additionally, the detailed registry data allowed us to follow women from early age into adulthood, providing a more comprehensive understanding of the long-term effects of these conditions.

However, there are several limitations to our study. Firstly, our analysis only includes women with diagnosed endometriosis and PVD recorded in the National Patient Registry, which only covers diagnoses made by physicians in health care specialties, mainly gynecologists. Women with undiagnosed endometriosis and PVD, as well as those diagnosed in primary care or other health clinics, or women diagnosed prior to 1997, may not be captured in our analysis. Thus, it is possible that some women in the control group are, in fact, suffering from endometriosis and/or PVD. This would, however, most likely lead to a downward bias in our estimates, meaning that the true effect is underestimated. Furthermore, women who do not seek treatment or who experience a delay in diagnosis may be underrepresented in our sample.

Additionally, we lack data on the underlying diagnoses for sickness absence, which may be influenced by concurrent chronic pain and/or psychiatric disorders. This is particularly relevant for women with PVD, as their pain is often provoked by sexual activity rather than being constant. Finally, our study does not investigate the cause of infertility in women with PVD, as this aspect falls beyond the scope of our research.

In conclusion, while our study benefits from the use of high-quality registry data and a large sample size, there are limitations to our analysis that must be taken into consideration when interpreting our results. Further research is needed to fully understand the impact of endometriosis and PVD on women’s health and labor market outcomes.

## Conclusions

Our study shows that women with diagnosed endometriosis and/or PVD experience a range of adverse health outcomes. These include reduced fertility, worse labor market outcomes, and a higher prevalence of mental health and concurrent pain comorbidities. Furthermore, our findings suggest that these negative effects may start as early as the early teens, around the time of menarche, as indicated by the escalation of in- and out-patient visits. Taken together, these results highlight the importance of early recognition and treatment of endometriosis and/or PVD. By addressing these conditions in a timely manner, we may be able to improve the general health and quality of life of affected women, as well as their ability to participate in the labor market.

## Supporting information

S1 File Included diagnoses, cases and comorbidity. (DOCX)

S1 Fig Results from descriptive raw data (all groups). (a) In-patient visits (b) Out-patient visits (c) Earnings (SEK) (d) Income from sickness benefits (SEK) (e) Income from unemployment benefits (SEK). Inpatients visits, age 0–43. Out-patient visits and labor market outcomes, age 6–43.
(ZIP)

S2 Fig Results from regression analyses of in- and outpatient visits and labor market outcomes (all groups). (a) In-patient visits (b) Out-patient visits (c) Earnings (SEK) (d) Income from sickness benefits (SEK) (e) Income from unemployment benefits (SEK). Coefficient plots with point estimates and 95% confidence interval. Black line represents control women.
(ZIP)
