# Supplementary material for: Health and socioeconomic well-being of women with endometriosis and provoked vestibulodynia: Longitudinal insights from Swedish registry data
Source: PLoS One. 2024 Sep 3;19(9):e0307412. doi: 10.1371/journal.pone.0307412 (PMC11371220; doi:10.1371/journal.pone.0307412)
Supplement: S1 File — (DOCX) [file pone.0307412.s001.docx]

**Table A1.** ICD-codes and variable definition.

|  | **ICD-10** |
| --- | --- |
| **Cases** |  |
| Provoked vestibulodynia (PVD) | N763 |
| Vaginismus | N942, F525 |
| Endometriosis | N80, N800, N801, N802, N803, N804, N805, N806, N808, N809  N978D |
| **Comorbidity** |  |
| **Composite index pain disorders** |  |
| ***Head*** |  |
| Migraine | G43 |
| Other headache | G44 |
| Headache | R51 |
| Inflammation in jaws | K102 |
| ***IBS*** | K58 |
| ***Muscles and joints*** |  |
| Pain in joint | M255 |
| Hypermobility syndrome | M357 |
| Back pain | M54 |
| Fibromyalgia | M797 |
| ***Bladder*** |  |
| Interstitial cystitis (chronic) | N301 |
| Urethritis | N34 |
| ***Unspecific pain*** | R52 |
| **Composite index psychiatric disorders** |  |
| Depression | F32 |
| Recurrent depression | F33 |
| Anxiety disorders | F41 |
| Obsessive compulsive disorders | F42 |
| Stress reactions | F43 |
| Eating disorders | F50 |
| Sleeping disorders | G47 |
| **Substance abuse** | F19 |
| **Neuropsychiatric disorders** |  |
| Asperger’s syndrome | F845 |
| Hyperkinetic disorder | F90 |
|  |  |
|  | **Medical Birth Registry** |
| **In vitro fertilization** | OFRIICSI |
|  | OFRIABEF |
|  |  |

**Figure A1**

**
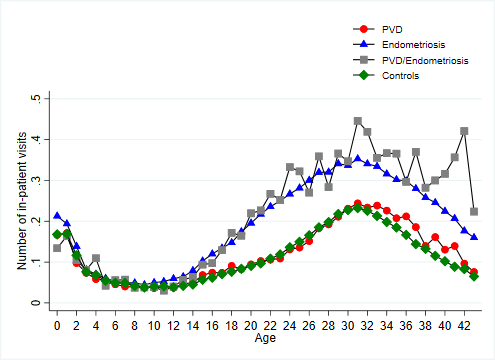

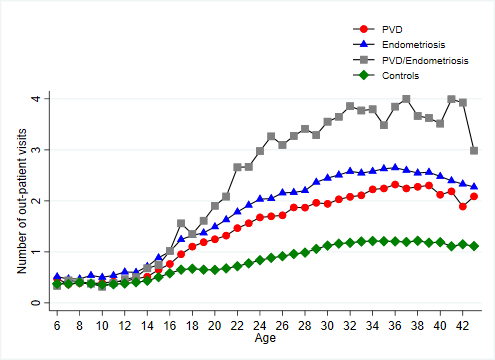

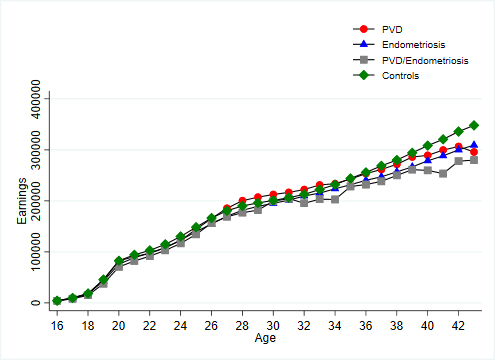

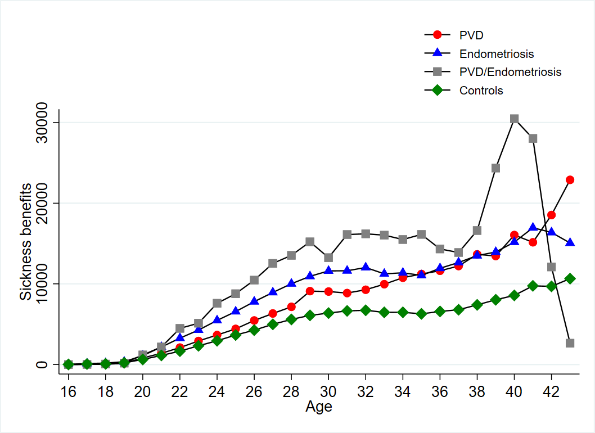

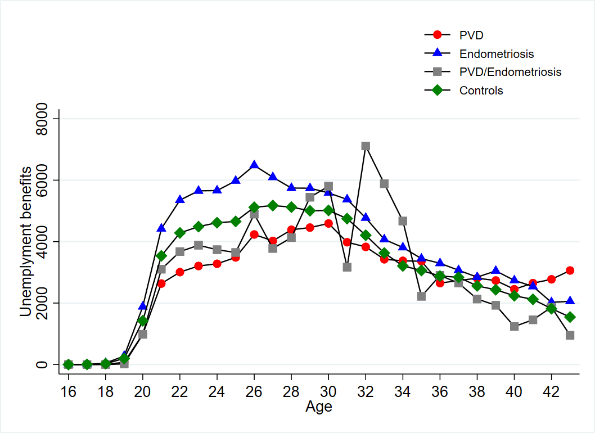
**

**Figure A2**

**
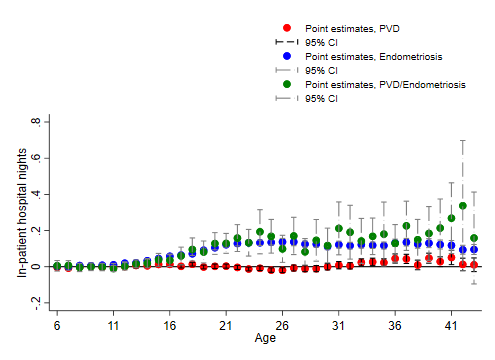

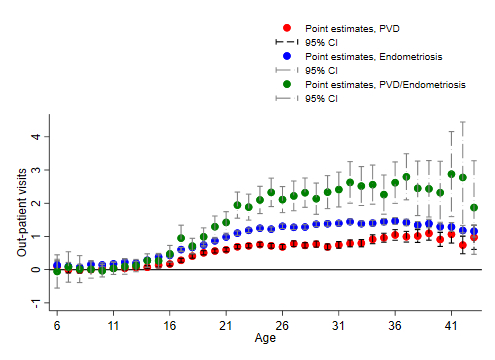

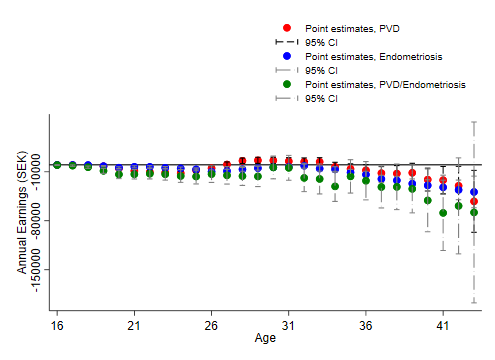

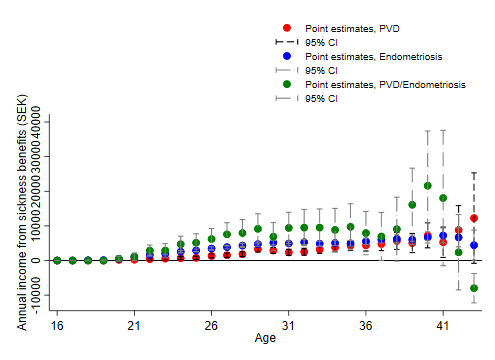

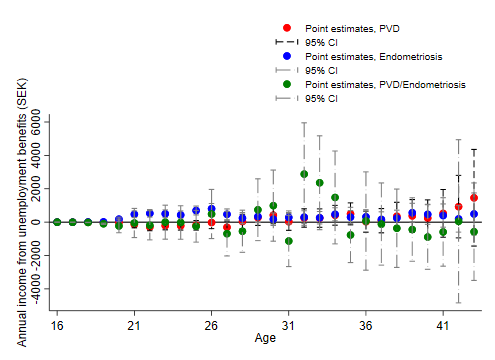
**
