# Supplementary figures and images for: Health and socioeconomic well-being of women with endometriosis and provoked vestibulodynia: Longitudinal insights from Swedish registry data
Source: PLoS One. 2024 Sep 3;19(9):e0307412. doi: 10.1371/journal.pone.0307412 (PMC11371220; doi:10.1371/journal.pone.0307412)

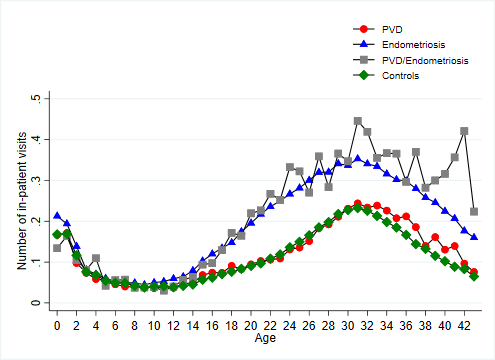

Supplement: S1 Fig — (a) In-patient visits (b) Out-patient visits (c) Earnings (SEK) (d) Income from sickness benefits (SEK) (e) Income from unemployment benefits (SEK). Inpatients visits, age 0–43. Out-patient visits and labor market outcomes, age 6–43. (ZIP) [file pone.0307412.s002.zip › Fig S1a.tif]

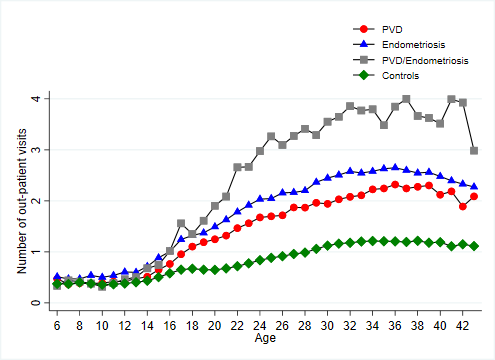

Supplement: S1 Fig — (a) In-patient visits (b) Out-patient visits (c) Earnings (SEK) (d) Income from sickness benefits (SEK) (e) Income from unemployment benefits (SEK). Inpatients visits, age 0–43. Out-patient visits and labor market outcomes, age 6–43. (ZIP) [file pone.0307412.s002.zip › Fig S1b.tif]

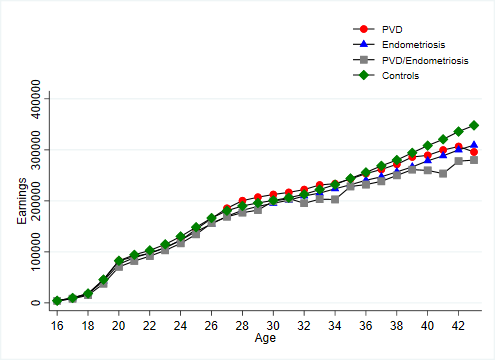

Supplement: S1 Fig — (a) In-patient visits (b) Out-patient visits (c) Earnings (SEK) (d) Income from sickness benefits (SEK) (e) Income from unemployment benefits (SEK). Inpatients visits, age 0–43. Out-patient visits and labor market outcomes, age 6–43. (ZIP) [file pone.0307412.s002.zip › Fig S1c.tif]

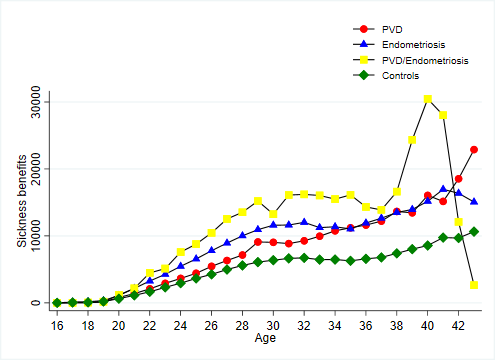

Supplement: S1 Fig — (a) In-patient visits (b) Out-patient visits (c) Earnings (SEK) (d) Income from sickness benefits (SEK) (e) Income from unemployment benefits (SEK). Inpatients visits, age 0–43. Out-patient visits and labor market outcomes, age 6–43. (ZIP) [file pone.0307412.s002.zip › Fig S1d.tif]

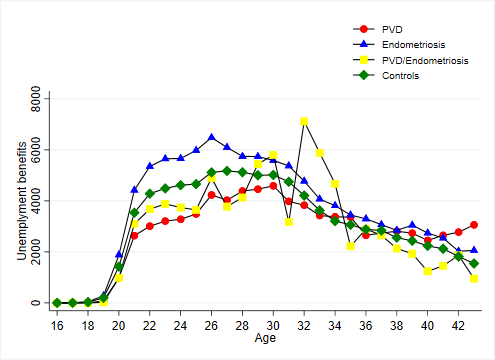

Supplement: S1 Fig — (a) In-patient visits (b) Out-patient visits (c) Earnings (SEK) (d) Income from sickness benefits (SEK) (e) Income from unemployment benefits (SEK). Inpatients visits, age 0–43. Out-patient visits and labor market outcomes, age 6–43. (ZIP) [file pone.0307412.s002.zip › Fig S1e.tif]

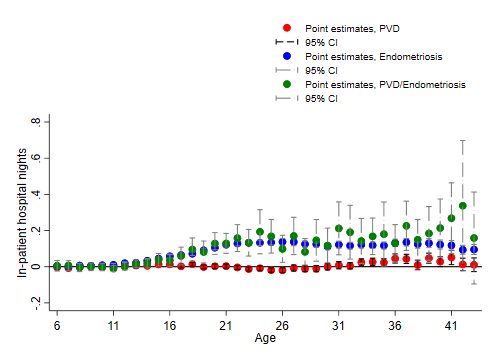

Supplement: S2 Fig — (a) In-patient visits (b) Out-patient visits (c) Earnings (SEK) (d) Income from sickness benefits (SEK) (e) Income from unemployment benefits (SEK). Coefficient plots with point estimates and 95% confidence interval. Black line represents control women. (ZIP) [file pone.0307412.s003.zip › Fig S2a.tif]

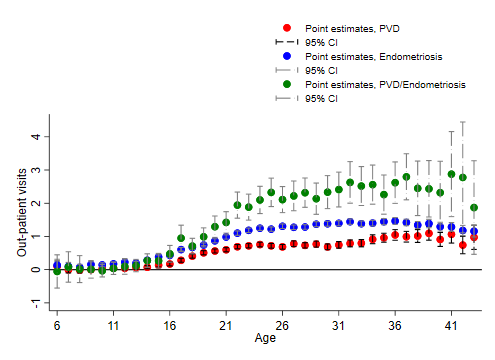

Supplement: S2 Fig — (a) In-patient visits (b) Out-patient visits (c) Earnings (SEK) (d) Income from sickness benefits (SEK) (e) Income from unemployment benefits (SEK). Coefficient plots with point estimates and 95% confidence interval. Black line represents control women. (ZIP) [file pone.0307412.s003.zip › Fig S2b.tif]

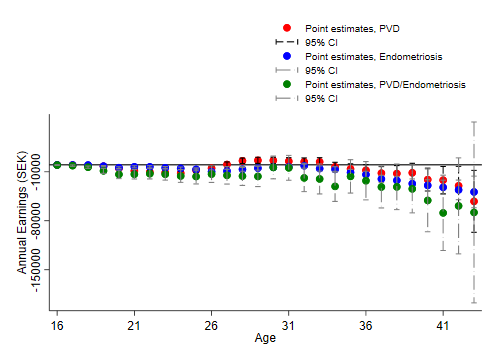

Supplement: S2 Fig — (a) In-patient visits (b) Out-patient visits (c) Earnings (SEK) (d) Income from sickness benefits (SEK) (e) Income from unemployment benefits (SEK). Coefficient plots with point estimates and 95% confidence interval. Black line represents control women. (ZIP) [file pone.0307412.s003.zip › Fig S2c.tif]

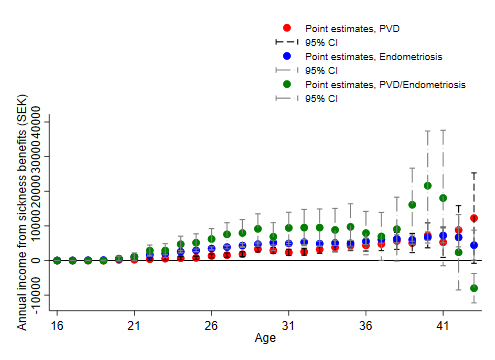

Supplement: S2 Fig — (a) In-patient visits (b) Out-patient visits (c) Earnings (SEK) (d) Income from sickness benefits (SEK) (e) Income from unemployment benefits (SEK). Coefficient plots with point estimates and 95% confidence interval. Black line represents control women. (ZIP) [file pone.0307412.s003.zip › Fig S2d.tif]

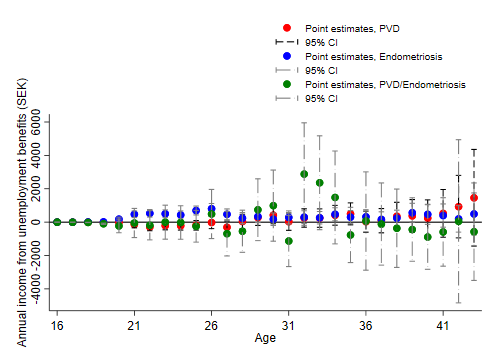

Supplement: S2 Fig — (a) In-patient visits (b) Out-patient visits (c) Earnings (SEK) (d) Income from sickness benefits (SEK) (e) Income from unemployment benefits (SEK). Coefficient plots with point estimates and 95% confidence interval. Black line represents control women. (ZIP) [file pone.0307412.s003.zip › Fig S2e.tif]
