# Supplementary material for: Health and socioeconomic well-being of women with endometriosis and provoked vestibulodynia: Longitudinal insights from Swedish registry data
Source: PLoS One. 2024 Sep 3;19(9):e0307412. doi: 10.1371/journal.pone.0307412 (PMC11371220; doi:10.1371/journal.pone.0307412)
Supplement: Attachment — Submitted filename: Response to reviewer.docx [file pone.0307412.s004.docx]

***Resubmission of “Health and Socioeconomic Well-being of Women with Endometriosis and Provoked Vestibulodynia: Longitudinal Insights from Swedish Registry Data***

**Journal requirements:**

When submitting your revision, we need you to address these additional requirements.

1. Please ensure that your manuscript meets PLOS ONE's style requirements, including those for file naming.

>>>The manuscript meets PLOS ONE's style requirements.

2. We note that the grant information you provided in the ‘Funding Information’ and ‘Financial Disclosure’ sections do not match. When you resubmit, please ensure that you provide the correct grant numbers for the awards you received for your study in the ‘Funding Information’ section.

>>> We ensure that the correct grant number is provided in the ‘Funding Information’ section, “The study was supported by a grant from by the Swedish Social Insurance Agency, Dnr 052666-17”.

3. We note that you have indicated that there are restrictions to data sharing for this study. PLOS only allows data to be available upon request if there are legal or ethical restrictions on sharing data publicly.

>>> Data availability statement: This paper uses data from Statistics Sweden and the Swedish National Board of Health and Welfare. Because the data contain sensitive information on individuals, the Swedish law requires that users of the data obtain permission from the Swedish Ethical Review Authority that could be contacted at [registrator@etikprovning.se](mailto:registrator@etikprovning.se).

4. Please review your reference list to ensure that it is complete and correct.

>>> The reference list is complete and correct. Some changes of references have been made on request by the reviewer. Please see below.

**Response to reviewer**

Thank you for valuable feedback on out manuscript *“Health and Socioeconomic Well-being of Women with Endometriosis and Provoked Vestibulodynia: Longitudinal Insights from Swedish Registry Data”.*

In the following text we have answered all the comments from the reviewer and also included where in the manuscript changes have been made.

Reviewer #1: Manuscript Title: Health and Socioeconomic Well-being of Women with Endometriosis and Provoked Vestibulodynia: Longitudinal Insights from Swedish Registry Data

Let me first congratulate the authors for writing this article. Chronic genital pain in women with the related diseases such as endometriosis and vestibulodynia, cause debilitating conditions in young women and have a negative effect on the quality of their social and professional life as well as their fertility. I have some comments.

>>> We want to extend our gratitude to the reviewer for helpful and insightful comments and feedback on our manuscript.

1- Although this data is valuable, but please the authors explain why your information is reported until 2018?

>>> Thank you for your question. This study is part of a larger project on chronic pelvic pain that began in 2018. The data acquisition process is inherently slow, as it requires thorough ethical vetting by the Swedish Ethical Authority, followed by the application for and integration of data from multiple Swedish registries (obtained from multiple sources Statistics Sweden and the National Board of Health and Welfare). Our final dataset was fully completed in 2020. Moreover, our project was unfortunately delayed by the covid19 pandemic.

2- Diagnostic criteria of endometriosis and vestibulodynia should be described with reference in the method part.

>>> Thank you for your advice. Please see revised version, page 6 and 7.

3- All items in Table 1 should be described in the method part (such as Wage earnings, Sickness benefits and Unemployment benefits.

>>> Thank you for your suggestion. We have rewritten and made some clarifications in the section on “Outcomes of interest”, page 7 and added the missing variables in Table 1. Please see revised version, page 7-8 and Table 1.

4- The basic demographic data such as age, BMI and marital status should be mentioned in the results part.

>>> Thank you for your suggestion. We have added information on birth year and marital status to the result section (and Table 1). Unfortunately, we do not have information on BMI. Additionally, to better illustrate labor market productivity across groups, we have focused on labor market outcomes at age 35 instead of the mean income (for all ages). Please see revised version in Table 1, page 11.

5- All abbreviation such as IVF should be described below the tables.

>>> Thank you for your comment. Please see revised Table 1 and 2 legend, page 13 and 16.

6- Table 1 needs p value to compare the mentioned characteristics between groups.

>>> Thank you for your comment. However, Table 1 provides simple summary statistics and does not include any statistical testing, so we do not have any p-values to present for this table. In Table 2, we examine differences in outcomes using regression analysis and present both confidence intervals and p-values. We have merged the descriptive part of the results with the regression results to improve clarity of our findings. Please see result section, page 9-11.

7- Most references are outdated and need to be updated.

>>> Thank you for your advice. We have not removed any references but added some newer ones. Please see revised introduction and reference list.

>>> Thank you very much again for your comments and feedback. We highly appreciate your assistance.
