# Supplementary material for: Health and socioeconomic well-being of women with endometriosis and provoked vestibulodynia: Longitudinal insights from Swedish registry data
Source: PLoS One. 2024 Sep 3;19(9):e0307412. doi: 10.1371/journal.pone.0307412 (PMC11371220; doi:10.1371/journal.pone.0307412)
Supplement: Peer review history

# Peer review: Decision Letter 0

**DOI:** 10.1371/journal.pone.0307412.r001

**Authors:** Ali Montazeri

15 May 2024

PONE-D-23-42595Health and Socioeconomic Well-being of Women with Endometriosis and Provoked Vestibulodynia:Longitudinal Insights from Swedish Registry DataPLOS ONE

Dear Dr. Bohm-Starke,

Thank you for submitting your manuscript to PLOS ONE. After careful consideration, we feel that it has merit but does not fully meet PLOS ONE’s publication criteria as it currently stands. Therefore, we invite you to submit a revised version of the manuscript that addresses the points raised during the review process. Specifically I would like to remind you to update the citations and perhaps looking on papers on quality of life in this patient population.

Please submit your revised manuscript by Jun 29 2024 11:59PM. If you will need more time than this to complete your revisions, please reply to this message or contact the journal office at plosone@plos.org. When you're ready to submit your revision, log on to https://www.editorialmanager.com/pone/ and select the 'Submissions Needing Revision' folder to locate your manuscript file.

Please include the following items when submitting your revised manuscript:

- A rebuttal letter that responds to each point raised by the academic editor and reviewer(s). You should upload this letter as a separate file labeled 'Response to Reviewers'.
- A marked-up copy of your manuscript that highlights changes made to the original version. You should upload this as a separate file labeled 'Revised Manuscript with Track Changes'.
- An unmarked version of your revised paper without tracked changes. You should upload this as a separate file labeled 'Manuscript'.

If you would like to make changes to your financial disclosure, please include your updated statement in your cover letter. Guidelines for resubmitting your figure files are available below the reviewer comments at the end of this letter.

If applicable, we recommend that you deposit your laboratory protocols in protocols.io to enhance the reproducibility of your results. Protocols.io assigns your protocol its own identifier (DOI) so that it can be cited independently in the future. For instructions see: https://journals.plos.org/plosone/s/submission-guidelines#loc-laboratory-protocols. Additionally, PLOS ONE offers an option for publishing peer-reviewed Lab Protocol articles, which describe protocols hosted on protocols.io. Read more information on sharing protocols at https://plos.org/protocols?utm_medium=editorial-email&utm_source=authorletters&utm_campaign=protocols.

We look forward to receiving your revised manuscript.

Kind regards,

Ali Montazeri

Academic Editor

PLOS ONE

Journal requirements:

When submitting your revision, we need you to address these additional requirements.

1. Please ensure that your manuscript meets PLOS ONE's style requirements, including those for file naming. The PLOS ONE style templates can be found at

https://journals.plos.org/plosone/s/file?id=wjVg/PLOSOne_formatting_sample_main_body.pdf and

https://journals.plos.org/plosone/s/file?id=ba62/PLOSOne_formatting_sample_title_authors_affiliations.pdf

2. We note that the grant information you provided in the ‘Funding Information’ and ‘Financial Disclosure’ sections do not match. 

When you resubmit, please ensure that you provide the correct grant numbers for the awards you received for your study in the ‘Funding Information’ section.

3. We note that you have indicated that there are restrictions to data sharing for this study. PLOS only allows data to be available upon request if there are legal or ethical restrictions on sharing data publicly. For more information on unacceptable data access restrictions, please see http://journals.plos.org/plosone/s/data-availability#loc-unacceptable-data-access-restrictions. 

Before we proceed with your manuscript, please address the following prompts:

a) If there are ethical or legal restrictions on sharing a de-identified data set, please explain them in detail (e.g., data contain potentially identifying or sensitive patient information, data are owned by a third-party organization, etc.) and who has imposed them (e.g., a Research Ethics Committee or Institutional Review Board, etc.). Please also provide contact information for a data access committee, ethics committee, or other institutional body to which data requests may be sent.

b) If there are no restrictions, please upload the minimal anonymized data set necessary to replicate your study findings to a stable, public repository and provide us with the relevant URLs, DOIs, or accession numbers. For a list of recommended repositories, please see

https://journals.plos.org/plosone/s/recommended-repositories. You also have the option of uploading the data as Supporting Information files, but we would recommend depositing data directly to a data repository if possible.

We will update your Data Availability statement on your behalf to reflect the information you provide.

4. Please review your reference list to ensure that it is complete and correct. If you have cited papers that have been retracted, please include the rationale for doing so in the manuscript text, or remove these references and replace them with relevant current references. Any changes to the reference list should be mentioned in the rebuttal letter that accompanies your revised manuscript. If you need to cite a retracted article, indicate the article’s retracted status in the References list and also include a citation and full reference for the retraction notice.

Additional Editor Comments:

[Note: HTML markup is below. Please do not edit.]

Reviewers' comments:

Reviewer's Responses to Questions

**Comments to the Author**

1. Is the manuscript technically sound, and do the data support the conclusions?

The manuscript must describe a technically sound piece of scientific research with data that supports the conclusions. Experiments must have been conducted rigorously, with appropriate controls, replication, and sample sizes. The conclusions must be drawn appropriately based on the data presented. 

Reviewer #1: Yes

********** 

2. Has the statistical analysis been performed appropriately and rigorously? 

Reviewer #1: Yes

********** 

3. Have the authors made all data underlying the findings in their manuscript fully available?

The PLOS Data policy requires authors to make all data underlying the findings described in their manuscript fully available without restriction, with rare exception (please refer to the Data Availability Statement in the manuscript PDF file). The data should be provided as part of the manuscript or its supporting information, or deposited to a public repository. For example, in addition to summary statistics, the data points behind means, medians and variance measures should be available. If there are restrictions on publicly sharing data—e.g. participant privacy or use of data from a third party—those must be specified.

Reviewer #1: Yes

********** 

4. Is the manuscript presented in an intelligible fashion and written in standard English?

PLOS ONE does not copyedit accepted manuscripts, so the language in submitted articles must be clear, correct, and unambiguous. Any typographical or grammatical errors should be corrected at revision, so please note any specific errors here.

Reviewer #1: Yes

********** 

5. Review Comments to the Author

Please use the space provided to explain your answers to the questions above. You may also include additional comments for the author, including concerns about dual publication, research ethics, or publication ethics. (Please upload your review as an attachment if it exceeds 20,000 characters)

Reviewer #1: Manuscript Title: Health and Socioeconomic Well-being of Women with Endometriosis and Provoked Vestibulodynia: Longitudinal Insights from Swedish Registry Data

Let me first congratulate the authors for writing this article. Chronic genital pain in women with the related diseases such as endometriosis and vestibulodynia, cause debilitating conditions in young women and have a negative effect on the quality of their social and professional life as well as their fertility. I have some comments.

1- Although this data is valuable, but please the authors explain why your information is reported until 2018?

2- Diagnostic criteria of endometriosis and vestibulodynia should be described with reference in the method part.

3- All items in Table 1 should be described in the method part (such as Wage earnings, Sickness benefits and Unemployment benefits.

4- The basic demographic data such as age, BMI and marital status should be mentioned in the results part.

5- All abbreviation such as IVF should be described below the tables.

6- Table 1 needs p value to compare the mentioned characteristics between groups.

7- Most references are outdated and need to be updated.

Best regards,

********** 

6. PLOS authors have the option to publish the peer review history of their article (what does this mean?). If published, this will include your full peer review and any attached files.

If you choose “no”, your identity will remain anonymous but your review may still be made public.

**Do you want your identity to be public for this peer review?** For information about this choice, including consent withdrawal, please see our Privacy Policy.

Reviewer #1: No

**********

[NOTE: If reviewer comments were submitted as an attachment file, they will be attached to this email and accessible via the submission site. Please log into your account, locate the manuscript record, and check for the action link "View Attachments". If this link does not appear, there are no attachment files.]

While revising your submission, please upload your figure files to the Preflight Analysis and Conversion Engine (PACE) digital diagnostic tool, https://pacev2.apexcovantage.com/. PACE helps ensure that figures meet PLOS requirements. To use PACE, you must first register as a user. Registration is free. Then, login and navigate to the UPLOAD tab, where you will find detailed instructions on how to use the tool. If you encounter any issues or have any questions when using PACE, please email PLOS at figures@plos.org. Please note that Supporting Information files do not need this step.

# Author comment: Author response to Decision Letter 0

**DOI:** 10.1371/journal.pone.0307412.r002

23 Jun 2024

Resubmission of “Health and Socioeconomic Well-being of Women with Endometriosis and Provoked Vestibulodynia: Longitudinal Insights from Swedish Registry Data

Journal requirements:

When submitting your revision, we need you to address these additional requirements.

1. Please ensure that your manuscript meets PLOS ONE's style requirements, including those for file naming. 

>>>The manuscript meets PLOS ONE's style requirements. 

2. We note that the grant information you provided in the ‘Funding Information’ and ‘Financial Disclosure’ sections do not match. When you resubmit, please ensure that you provide the correct grant numbers for the awards you received for your study in the ‘Funding Information’ section.

>>> We ensure that the correct grant number is provided in the ‘Funding Information’ section, “The study was supported by a grant from by the Swedish Social Insurance Agency, Dnr 052666-17”.

3. We note that you have indicated that there are restrictions to data sharing for this study. PLOS only allows data to be available upon request if there are legal or ethical restrictions on sharing data publicly. 

>>> Data availability statement: This paper uses data from Statistics Sweden and the Swedish National Board of Health and Welfare. Because the data contain sensitive information on individuals, the Swedish law requires that users of the data obtain permission from the Swedish Ethical Review Authority. The authors are willing to assist anyone who wishes to access this data set.

4. Please review your reference list to ensure that it is complete and correct. 

>>> The reference list is complete and correct. Some changes of references have been made on request by the reviewer. Please see below. 

Response to reviewer

Thank you for valuable feedback on out manuscript “Health and Socioeconomic Well-being of Women with Endometriosis and Provoked Vestibulodynia: Longitudinal Insights from Swedish Registry Data”.

In the following text we have answered all the comments from the reviewer and also included where in the manuscript changes have been made.

Reviewer #1: Manuscript Title: Health and Socioeconomic Well-being of Women with Endometriosis and Provoked Vestibulodynia: Longitudinal Insights from Swedish Registry Data

Let me first congratulate the authors for writing this article. Chronic genital pain in women with the related diseases such as endometriosis and vestibulodynia, cause debilitating conditions in young women and have a negative effect on the quality of their social and professional life as well as their fertility. I have some comments.

>>> We want to extend our gratitude to the reviewer for helpful and insightful comments and feedback on our manuscript. 

1- Although this data is valuable, but please the authors explain why your information is reported until 2018?

>>> Thank you for your question. This study is part of a larger project on chronic pelvic pain that began in 2018. The data acquisition process is inherently slow, as it requires thorough ethical vetting by the Swedish Ethical Authority, followed by the application for and integration of data from multiple Swedish registries (obtained from multiple sources Statistics Sweden and the National Board of Health and Welfare). Our final dataset was fully completed in 2020. Moreover, our project was unfortunately delayed by the covid19 pandemic. 

2- Diagnostic criteria of endometriosis and vestibulodynia should be described with reference in the method part.

>>> Thank you for your advice. Please see revised version, page 6 and 7. 

3- All items in Table 1 should be described in the method part (such as Wage earnings, Sickness benefits and Unemployment benefits.

>>> Thank you for your suggestion. We have rewritten and made some clarifications in the section on “Outcomes of interest”, page 7 and added the missing variables in Table 1. Please see revised version, page 7-8 and Table 1. 

4- The basic demographic data such as age, BMI and marital status should be mentioned in the results part.

>>> Thank you for your suggestion. We have added information on birth year and marital status to the result section (and Table 1). Unfortunately, we do not have information on BMI. Additionally, to better illustrate labor market productivity across groups, we have focused on labor market outcomes at age 35 instead of the mean income (for all ages). Please see revised version in Table 1, page 11. 

5- All abbreviation such as IVF should be described below the tables.

>>> Thank you for your comment. Please see revised Table 1 and 2 legend, page 13 and 16. 

6- Table 1 needs p value to compare the mentioned characteristics between groups.

>>> Thank you for your comment. However, Table 1 provides simple summary statistics and does not include any statistical testing, so we do not have any p-values to present for this table. In Table 2, we examine differences in outcomes using regression analysis and present both confidence intervals and p-values. We have merged the descriptive part of the results with the regression results to improve clarity of our findings. Please see result section, page 9-11. 

7- Most references are outdated and need to be updated.

>>> Thank you for your advice. We have not removed any references but added some newer ones. Please see revised introduction and reference list. 

>>> Thank you very much again for your comments and feedback. We highly appreciate your assistance.

Attachment Submitted filename: Response to reviewer.docx

# Editor report: Decision Letter 1

**DOI:** 10.1371/journal.pone.0307412.r003

**Authors:** Ali Montazeri

4 Jul 2024

Health and Socioeconomic Well-being of Women with Endometriosis and Provoked Vestibulodynia:Longitudinal Insights from Swedish Registry Data

PONE-D-23-42595R1

Dear Dr. Bohm-Starke ,

We’re pleased to inform you that your manuscript has been judged scientifically suitable for publication and will be formally accepted for publication once it meets all outstanding technical requirements.

Within one week, you’ll receive an e-mail detailing the required amendments. When these have been addressed, you’ll receive a formal acceptance letter and your manuscript will be scheduled for publication.

An invoice will be generated when your article is formally accepted. Please note, if your institution has a publishing partnership with PLOS and your article meets the relevant criteria, all or part of your publication costs will be covered. Please make sure your user information is up-to-date by logging into Editorial Manager at Editorial Manager® and clicking the ‘Update My Information' link at the top of the page. If you have any questions relating to publication charges, please contact our Author Billing department directly at authorbilling@plos.org.

If your institution or institutions have a press office, please notify them about your upcoming paper to help maximize its impact. If they’ll be preparing press materials, please inform our press team as soon as possible -- no later than 48 hours after receiving the formal acceptance. Your manuscript will remain under strict press embargo until 2 pm Eastern Time on the date of publication. For more information, please contact onepress@plos.org.

Kind regards,

Ali Montazeri

Academic Editor

PLOS ONE

Additional Editor Comments (optional):

Reviewers' comments:

# Editor report: Acceptance letter

**DOI:** 10.1371/journal.pone.0307412.r004

**Authors:** Ali Montazeri

24 Jul 2024

PONE-D-23-42595R1 

PLOS ONE

Dear Dr. Bohm-Starke, 

I'm pleased to inform you that your manuscript has been deemed suitable for publication in PLOS ONE. Congratulations! Your manuscript is now being handed over to our production team.

At this stage, our production department will prepare your paper for publication. This includes ensuring the following:

* All references, tables, and figures are properly cited

* All relevant supporting information is included in the manuscript submission,

* There are no issues that prevent the paper from being properly typeset

If revisions are needed, the production department will contact you directly to resolve them. If no revisions are needed, you will receive an email when the publication date has been set. At this time, we do not offer pre-publication proofs to authors during production of the accepted work. Please keep in mind that we are working through a large volume of accepted articles, so please give us a few weeks to review your paper and let you know the next and final steps. 

Lastly, if your institution or institutions have a press office, please let them know about your upcoming paper now to help maximize its impact. If they'll be preparing press materials, please inform our press team within the next 48 hours. Your manuscript will remain under strict press embargo until 2 pm Eastern Time on the date of publication. For more information, please contact onepress@plos.org.

If we can help with anything else, please email us at customercare@plos.org.

Thank you for submitting your work to PLOS ONE and supporting open access. 

Kind regards, 

PLOS ONE Editorial Office Staff

on behalf of

Professor Ali Montazeri 

Academic Editor

PLOS ONE
